# Protein kinase A regulatory subunit distribution in medulloblastoma

**DOI:** 10.1186/1471-2407-10-141

**Published:** 2010-04-14

**Authors:** Carla Mucignat-Caretta, Luca Denaro, Marco Redaelli, Domenico D'Avella, Antonio Caretta

**Affiliations:** 1 Department of Human Anatomy and Physiology, University of Padova, 35131 Padova - Italy; 2 Department of Neuroscience, University of Padova, 35131 Padova - Italy

## Abstract

**Background:**

Previous studies showed a differential distribution of the four regulatory subunits of cAMP-dependent protein kinases inside the brain, that changed in rodent gliomas: therefore, the distribution of these proteins inside the brain can give information on the functional state of the cells. Our goal was to examine human brain tumors to provide evidence for a differential distribution of protein kinase A in different tumors.

**Methods:**

The distribution of detergent insoluble regulatory (R1 and R2) and catalytic subunits of cAMP dependent kinases was examined in pediatric brain tumors by immunohistochemistry and fluorescent cAMP analogues binding.

**Results:**

R2 is organized in large single dots in medulloblastomas, while it has a different appearance in other tumors. Fluorescent cAMP labelling was observed only in medulloblastoma.

**Conclusions:**

A different distribution of cAMP dependent protein kinases has been observed in medulloblastoma.

## Background

Medulloblastoma (MB) is a tumor originating in the cerebellum, most often in children, peaking around seven years of age. Several histotypes can be recognized with different marker and prognosis. Medulloblastoma is thought to arise from the granule cell precursors, that during development reside in the external granule cell layer of the cerebellum. Among the most common modifications in MB are the presence of an isochromosome 17q (i17q) and disrupted development-related signalling pathways like Sonic Hehdgehog (SHH) and WNT [1]. It is therefore urgent to examine MB for anomalies in the biochemical phenotype, that may represent potential diagnostic landmarks or therapeutic targets. Until now, the search for specific mutations has lead to the identification of some changes that may be present in a percentage of patients. On the other hand, the functional dysregulation of intracellular pathways may be a common trait that deserves attention. The ubiquitous cAMP second messenger system involves the activation of different proteins, the most common being the cAMP-dependent protein kinase (PKA), a complex of two homodimers, that upon binding of two cAMP molecules to each regulatory subunit, releases the two catalytic subunits, that in turn phosphorylate their target proteins. In mammalian cells, four regulatory subunits (R1A, R1B, R2A, R2B) and two catalytic subunits (CA and CB) are known. The subunits share some common characteristics, but they differentially mediate a variety of cell functions, by segregating in subcellular compartments. PKA is involved in regulation of cell proliferation by acting on transcription factors, for example it may inhibit proliferation by uncoupling Ras from c-Raf activation [2]. The correct PKA cascade is necessary for the functional regulation of D-type cyclins, therefore a defective cAMP/PKA pathway may induce carcinogenesis in neuronal precursors [3]. This event may be influenced and even reverted by modifying the type of PKA subunit that is preferentially expressed by the cell [4]. Therefore the knowledge of modifications in PKA distribution or activity observed during development or transformation may be relevant to trace cell fate, being eventually targeted to modify cell phenotype. Different cAMP analogs have been already used to specifically address R1 and R2 in various diseases [5].

In the normal rodent brain, we described a differential distribution of PKA regulatory subunits, that changes during development and after chemical insults or surgical lesions. Our focus was on PKA bound to the cytoskeleton and organelles, since docking of PKA at particular sites can concentrate kinase phosphorylating activity in subcellular microdomains. The four different PKA regulatory subunits show a different appearance, time course and localization during rodent development. R1A appears postnatally and persists for two months in cerebellar nuclei, while in the archicerebellar granuli it appears from day 17 [6], a period in which Purkinje cells acquire their mature phenotype and express R1B [7]. Insoluble R2A is undetectable in the cerebellum, while R2B is present in the granule cells already at birth [8]. In addition, a different distribution of protein kinase regulatory subunits was observed in tumors of glial origin [9].

In the present paper we explored the possibility that human medulloblastoma specimens present a distribution of PKA different from the healthy cerebellar tissue, by analyzing tissue samples from various tumors.

## Methods

All reagents are from Sigma, Milan, Italy, unless otherwise stated. This study was approved by the Institutional Ethical Committee. Cases and diagnoses are summarized in Table 1. All patients were males. The three primitive medulloblastoma cases were all positive for synaptophysin and negative for GFAP. They were compared with four other tumors:

**Table 1:** Summary of cases

CASE	AGE	DIAGNOSIS
1	17 months	Anaplastic large cell medulloblastoma
2	9 years	Medulloblastoma, with focal anaplasia
3	4 years	Medulloblastoma
4	14 years	Medulloblastoma (at age 4), relapse Previous chemotherapy, no radiotherapy
5	17 years	Frontal glioblastoma following medulloblastoma at age 7 Previous chemotherapy and radiotherapy
6	2 years	ETANTR^b ^= embryonic tumor with abundant neuropil and true rosettes
7	17 years	Ewing's sarcoma (at age 15) relapse, extra-axial Previous chemotherapy and radiotherapy

- one MB relapse: this case was included in order to control for the replicability of results after therapy,

- one radio-induced glioblastoma following MB, to control for the specificity of MB results,

- one supratentorial embryonic tumor with abundant neuropil and true rosettes (ETANTR), which shares features of ependymoblastoma and neuroblastoma, to compare MB data with tumors originating probably from neural stem cells, different from granule cell precursors

- one extra-axial Ewing's sarcoma/PNET relapse, to control for a different primitive neuroectodermal tumor originating outside the central nervous system (see Tab. 1 for details).

Fresh tissue samples were collected during surgery and immediately snap-frozen in liquid nitrogen. They were conserved at -70°C until processing.

### Primary cultures

Fresh tissues were dissociated by shaking for 5 min in 0.25% Trypsin, 0.02% EDTA (1 ml/mm^3 ^tissue), then inactivated with complete high glucose Dulbecco Modified Eagle's Medium (DMEM), containing 10% Fetal Bovine Serum, 10 μg/ml streptomycin, 10 μg/ml tetracyclin, 100 I.U./ml penicillin, 25 μg/ml Plasmocin (InVivogen; Milan, Italy). The suspension was centrifuged at 37°C for 10 min at 1350 rpm. The supernatant was discharged and the pellet resuspended in 10 ml of DMEM. Cells were seeded for 48 hours, washed 3 times with PBS, and cultured in DMEM (as above, with only 25 μg/ml Plasmocin) for 3 weeks before freezing.

### Immunohistochemistry

After sectioning at 20 μm with a cryostat, sections were air-dried at room temperature, fixed for 1 hour in 5% formalin and incubated in Triton X-100 2% in Phosphate Buffered Saline (PBS, 10 mM phosphate, 150 mM NaCl, pH 7.4) for 30 minutes at 18°C. Alternative fixation procedures were: 1. one minute in 5% formalin at 37°C; 2. one minute in 5% formalin at 37°C followed by 2% Triton X-100 for 30 minutes at 18°C; 3. 30 minutes in 2% Triton X-100 at 18°C followed by one minute in 5% formalin, 1% Triton X-100 in PBS at 37°C. The rationale for using different fixation procedures stems from the fact that different fixation procedures interfere with the binding of proteins to membranes or cytoskeleton.

Sections were washed, dried and incubated overnight with primary antibodies.

Cells were thawed, fixed in formalin at 37°C for 1 minute and then incubated in 2% Triton X-100 in PBS for 5 minutes. Primary antibodies were incubated overnight.

The following antibodies were used: R1 (rabbit, sc-907, Santa Cruz Biotechnology, Santa Cruz, CA, USA) 1:200 on slices, 1:500 on cells; R2 (rabbit, sc-908, Santa Cruz Biotechnology) 1:200 on brain slices, 1:500 on cells; PKA catalytic subunit (rabbit, sc-903, Santa Cruz Biotechnology) 1:40 on slices, 1:200 on cells. The secondary antibody (anti-rabbit IgG Alexafluor 594 conjugate, Molecular Probes, Eugene, OR, USA) was incubated 30 minutes at 37°C (1:200 on tissue, 1:500 on cells). Cell nuclei were counterstained with bis-benzimide (Sigma).

Controls included: a positive control, mouse brain sections incubated with each of the above antibodies; negative controls omitting the primary antibody; for background staining the cells and tumors were incubated with normal rabbit serum.

For colocalization experiments, after immunohistochemistry, cells or tissue were incubated in the presence of Alexa 488-cAMP, Alexa 568-cAMP (Molecular Probes), or 8-thioacetamidofluorescein-cAMP (SAF-cAMP), which allow the visualization of R1A in the healthy brain tissue [6-8].

After immunohistochemistry, sections were counterstained with hematoxylin-eosin.

A Leica epifluorescence microscope (20×, 40×, 100× objectives) was used. Images (782 × 582 pixels) were captured with a color digital camera, using the same parameters within each experiment.

Contrast was enhanced with Corel Photo Paint by maximum 10% when necessary, at the same degree in both images if acquired with different filter sets. Panels were prepared with Corel Draw 12 (Corel Corporation, Ottawa, Canada).

## Results

Typical specific immounolabelling was present in the more conventional cold formalin fixation followed by Triton permeabilization, but the data were replicated also with the other fixation methods that were tried, albeit with different final intensities of the labelling, or with different background. Therefore, the data that will be presented refer to the former fixation protocol.

### PKA R2

**Figure 1:**
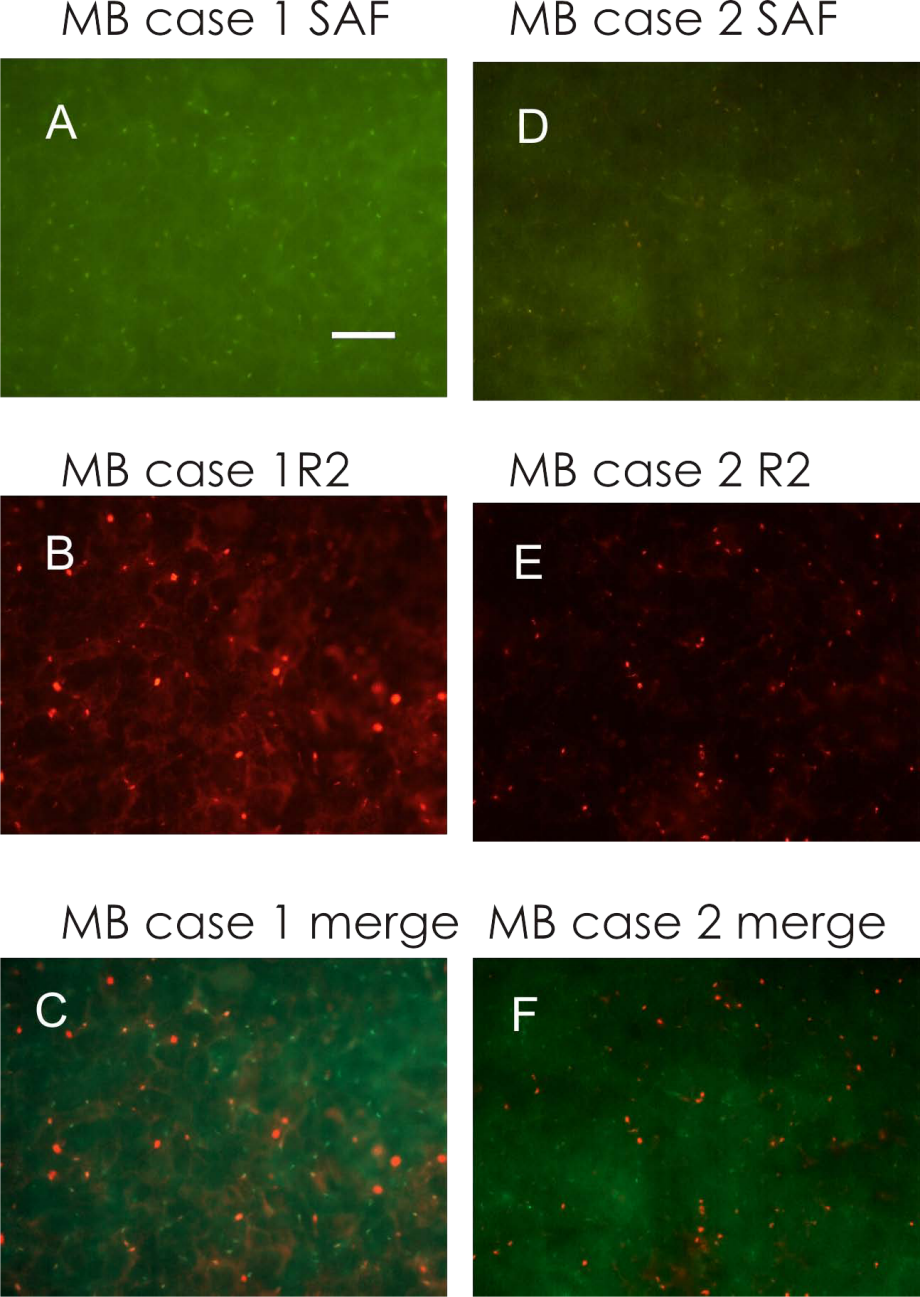
**Medulloblastoma cases 1 (A to C) and 2 (D to F)**. A: SAF-cAMP labelling. B: R2 immunolabelling. C: merge of A and B: SAF-cAMP labelling does not overlap to R2. D: SAF-cAMP labelling. E: R2 immunolabelling. F: merge of D and E: the two signals appear separated. Bar = 10 μm, 100× objective.

**Figure 2:**
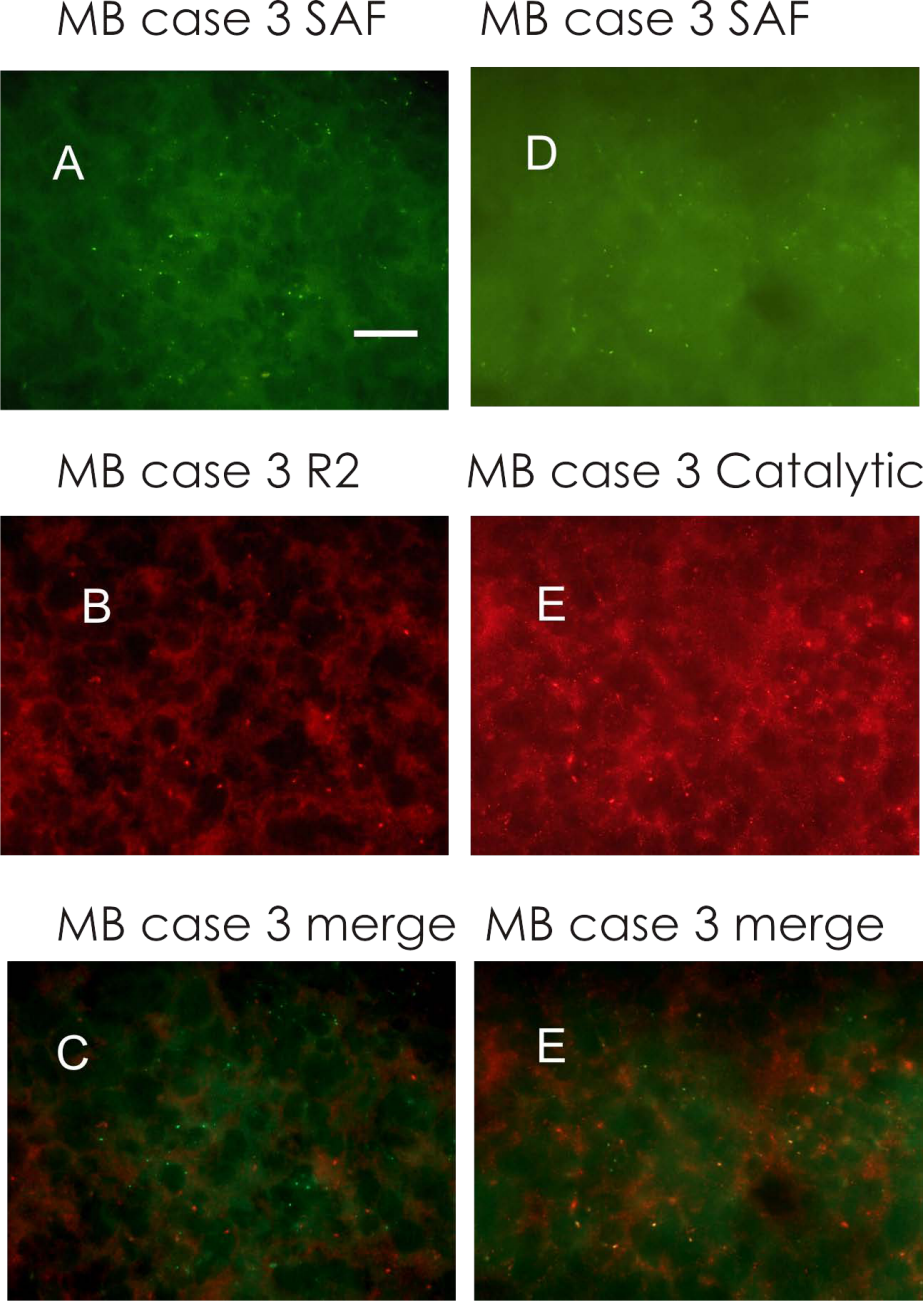
**Medulloblastoma, case 3**. A: SAF-cAMP labelling. B: R2 immunolabelling. C: merge of A and B, showing that SAF-cAMP does not overlap to R2. D: SAF-cAMP labelling. E: immunohistochemistry to reveal PKA catalytic subunit. F: merge of D and E: most of SAF-cAMP labelled structures are labelled also by antibody that reveals PKA catalytic subunit. Bar = 10 μm, 100× objective.

**Figure 3:**
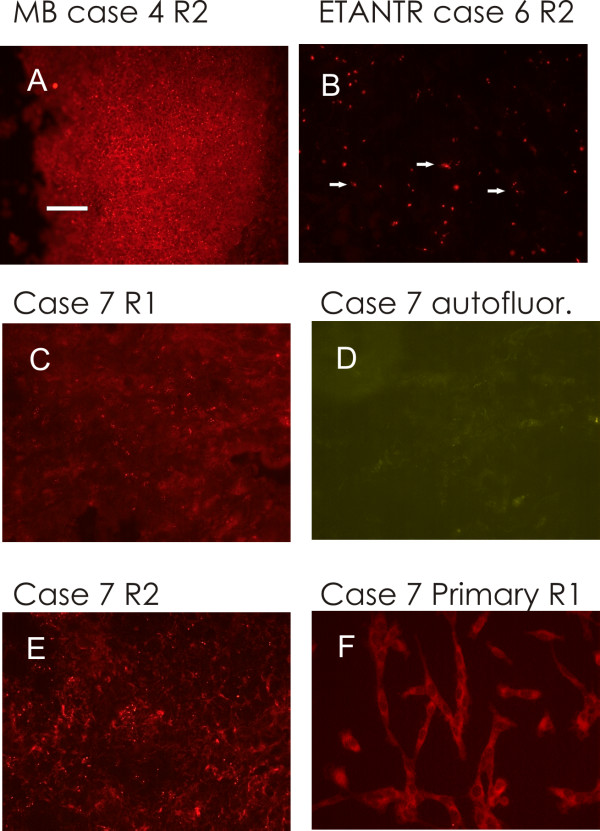
**Immunohistochemistry on different samples**. A: MB relapse, R2 immunolabelling, similar to primitive MB specimens, 40× objective. B: ETANTR, R2 immunolabelling (red). Arrows indicate three structures not seen in MB specimens. 100× objective. C to E: Ewing's sarcoma relapse. C and D: same field, 40× objective C: R1 immunolabelling in the form of fine dots; this kind of labelling was not present in MB samples. D: small autofluorescence granuli, albeit not numerous, were present in this specimen; autofluorescence was not present in primitive MB that did not undergo therapy. E: R2 immunolabelling, 20× objective. F: primary culture obtained from Ewing's sarcoma relapse; anti-R1 immunohistochemistry, 40× objective. Bar = 10 μm (B); bar = 25 μm (A, C, D, F); bar = 50 μm (E).

The three primitive MB and the MB relapse showed a moderate-intense labelling, in the form of isolated dots (Figs. 1B, E, 2B, E and 3A). A similar labelling was present also in Ewing's sarcoma relapse (Fig. 3E), while GBM was less intensely labelled. ETANTR showed a more varied types of labelling (Fig. 3B), since the R2 clusters could have different forms, not only round.

### PKA R1

The antibody against R1 did not show a dotted labelling. Only scanty elongated branches were present in all cases, except in Ewing's sarcoma, which showed clustered, finely dotted labelling (Fig. 3C). In the healthy brain of different species [7,8] as in human brain (Additional file 1: Fig. S1), we observed that 8-derivative fluorescently-tagged analogs of cAMP bind to the same protein clusters that are labelled by anti-PKA R1A antibodies, therefore we controlled for the fluorescent cAMP labelling in the same tumors. To our surprise, in the three primitive MB and in the MB relapse, the tissue showed numerous isolated small-to-medium size aggregates labelled by fluorescent cAMP analogs (Figs. 1A, D, 2A, D). The binding of fluorescent cAMP is specific, being abolished by 10 μM 8-Br-cAMP. These data points to a cAMP binding site which is different from those present in the healthy neurons, since it is not labelled by R1, nor by R2 antibodies (Figs. 1C, F, 2C), while being consistently labelled in a specific way by cAMP 8-derivatives. These data suggest that human MB cells present cAMP binding sites that are not normally present during development of granule precursor cells. Fluorescent cAMP labelling was not observed in Ewing's specimen, while very few fine and brilliant clusters were observed in few cells of ETANTR and GBM tissue, reminiscent of what is observed in healthy neurons of the rodent brain. Like in these cells, they do not overlap to R2-labelled structures.

### PKA Catalytic subunit

Catalytic subunit of PKA was present in MB specimens only, as numerous small, brilliant dots throughout the tissue (Fig. 2E). In some cases, these structures were labelled also with fluorescent cAMP (Fig. 2F): this suggests that complex structures containing both PKA catalytic subunit and a peculiar cAMP binding site, are present in MB tissue.

### Autofluorescence

Autofluorescence was present as enhanced background and some orange/yellow clusters, visible with both green and red filters, only in patients previously submitted to radiotherapy (for an example, see Fig. 3D). In every instance, autofluorescence was clearly distinguishable from secondary antibodies labelling by its broader emission spectrum that made it visible with different filter sets, both when particulate or present as a diffuse background.

### Cell cultures

To compare the present data with PKA R2 distribution that was observed in glioma models [9], cells obtained from tumor tissues were plated to obtain primary cultures, albeit MB primary cultures do not maintain some of the pathways present in the tumour [10]. No viable cultures were obtained from MB. GBM culture showed the same R2 clustered distribution restricted to the Golgi apparatus, as already described [9]. A similar restricted distribution was present in Ewing's sarcoma cultures, while R1 was present in the whole cytoplasm (Fig. 3F), confirming the presence of this subunit, as observed in the tissue.

## Discussion

The deregulation of signalling pathways is a landmark of cancer development.

The cAMP pathway may act on cell cycle, by activating PKA that translocates between subcellular compartments. By modulating the timing and localization of cAMP production, it is possible to affect cAMP effectors activation, that in turn act on the RAS/ERK and/or Hedgehog signalling pathways [11]. Therefore, targeting cAMP production is a viable strategy for supporting anticancer chemotherapy.

Several MB cell lines decrease their growth rate and can be differentiated by increasing cAMP levels [12]. Moreover, in a mouse model of MB, the genetic disruption of the PACAP gene, whose product activates the PKA pathway, drives cerebellar granule precursor cells to transformation and to the development of MB [13].

Different kinds of PKA involvement may be conceived, since the most common chromosomic alterations in medulloblastoma affect chromosome 17q and 7 [14], in which also the genes coding for PKA regulatory subunits R1A, R1B and R2B reside. On the other hand, during development Purkinje cells secrete Sonic Hedgehog factor, that induces granule cell precursors proliferation: this effect is inhibited by adenylate cyclase activation [15]. In the cerebellum, the chemokine receptor CXCR4 is a Gi-coupled receptor essential for the development of cerebellar cortex, that may act on SHH signalling through the control of cAMP [16]. Noteworthy, CXCR4 is highly expressed also in desmoplastic and nodular, but not classic, MB [17]. When its activity is blocked, and hence cAMP production increases, medulloblastoma growth is inhibited, an effect similar to phosphodiesterase blockade [18]. During development, PKA opposes the effects of Hedgehog proteins and effectors, therefore PKA can be a reasonable target for MB with a high Hedgehog activity. PKA activity is essential to prevent the expression of Hedgehog effectors, for example Gli, albeit no mutation was up to now detected in PKA genes R1A, R1B, R2B, CA and CB [19]. This may be due to the lethal effects of PKA mutations, given their widespread presence in every cell. No mutation is detected also in other proteins acting on transcription, like SHH, SMO, EN-2 that, similarly to R2B, are located on chromosome 7q, the most affected in MB [19].

The present data suggest that immunofluorescence techniques can be successfully applied to MB samples, given their low autofluorescence background, which is caused by accumulation of lipofuscin and other product of cell metabolism, and often prevents the use of fluorescence detection techniques in the brain of older animals and humans. In this way we could demonstrate that MB samples are characterized by a distinctive pattern of PKA regulatory subunit distribution inside the cell. While R2 could be detected in all samples, R1 could not be revealed using immunohistochemistry. On the other hand, MB samples but not the other tumors studied so far could be labelled with fluorescently-tagged cAMP, that in the normal brain of different species binds to clustered R1 subunit of PKA, present only in some types of differentiated neurons [7]. This observation suggests that MB present a cAMP-binding protein that is different, or differentially organised, from that already known. The present data may be useful for implementing differential diagnosis tools or to design potential therapeutic agents.

A further complication is added by the fact that each MB contains functionally heterogeneous cells, with a small fraction of them exhibiting stem-like properties [20]: the possibility exists that these cells show different signalling pathways dynamics, and hence can be targeted in a differential way.

Up to now it was postulated that cAMP formation results in PKA activation: however, the formation of cAMP-saturated PKA holoenzymes, which are inactive, has been reported under physiological conditions [21]. Future studies will cast light on the presence of cAMP and its effects on PKA activity and hence in MB cells cycling in vivo.

## Conclusions

The distribution of PKA regulatory subunits is modified in different tumors, suggesting that variations in common intracellular signalling pathways may be useful for differential diagnosis.

## Abbreviations

(C): Catalytic; (DMEM): Dulbecco Modified Eagle's Medium; (GFAP): Glial Fibrillary Acidic Protein; (PKA): cAMP dependent protein kinases; (R): regulatory subunits of cAMP dependent protein kinases; (SAF-cAMP): 8-thioacetamidofluorescein-cAMP; (SHH): Sonic Hedgehog.

## Competing interests

The authors declare that they have no competing interests.

## Authors' contributions

CMC and AC conceived the study, performed the immunohistochemistry, wrote the paper; MR performed the cell cultures; LD and DD provided the specimens and diagnosis. All authors have read and approved the final manuscript.

## Pre-publication history

The pre-publication history for this paper can be accessed here:

http://www.biomedcentral.com/1471-2407/10/141/prepub

## Supplementary Material

Additional file 1 **PKAR1/Alexa594-cAMP colocalization in the normal brain**. **FIGURE S1**. A section from normal brain (case 4) is shown, 100× objective, bar = 10 μm. A. immunohistochemistry for PKA R1, revealed with Alexa594 (red)-conjugated secondary antibody. B. the same section was then incubated with Alexa488 (green)-tagged cAMP. The arrows indicate two autofluorescent cells. C. Merge of A and B: the specific green signal overlaps with red signal. Click here for file
