# Supplementary figures and images for: Protein kinase A regulatory subunit distribution in medulloblastoma
Source: BMC Cancer. 2010 Apr 14;10:141. doi: 10.1186/1471-2407-10-141 (PMC2859386; doi:10.1186/1471-2407-10-141)

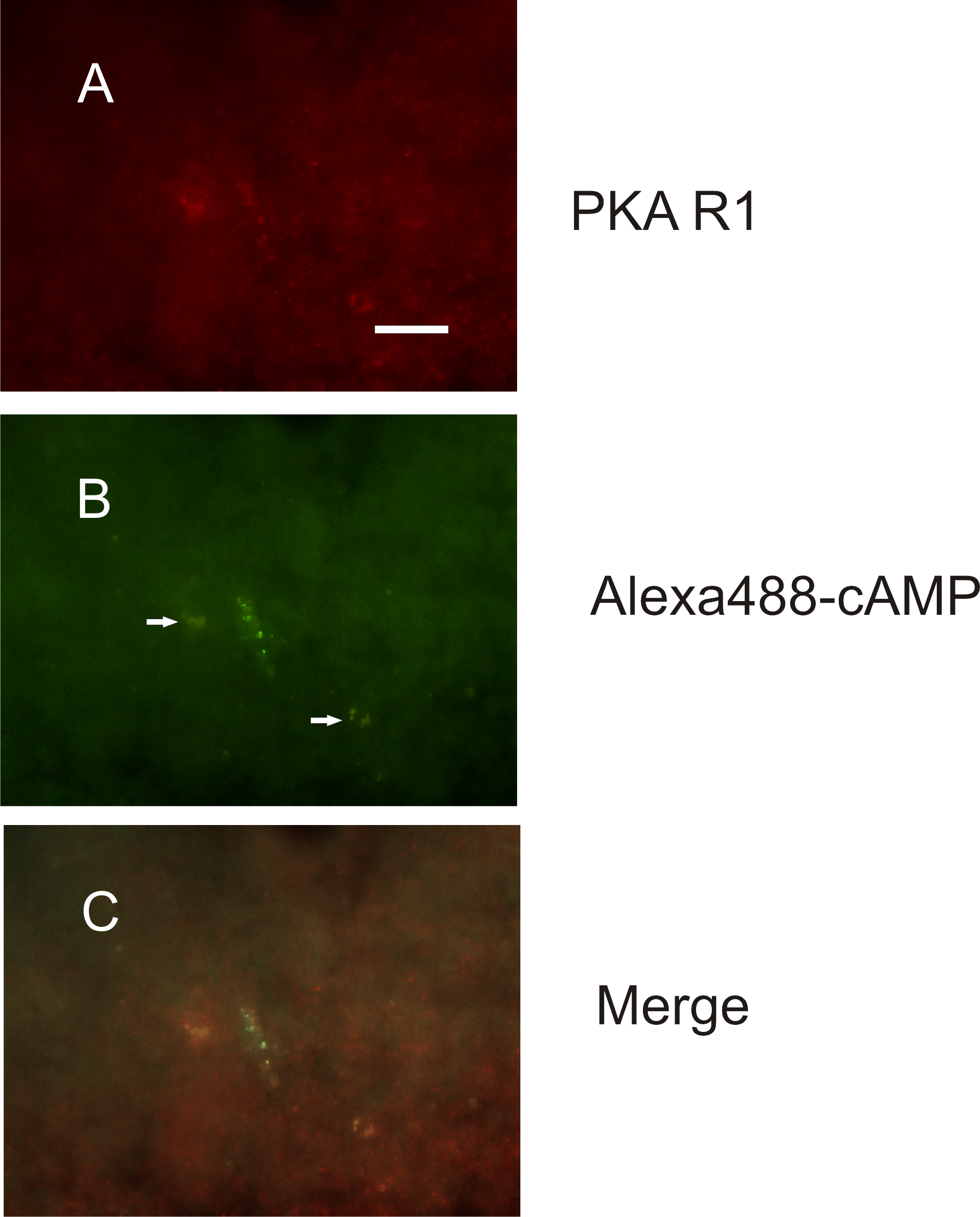

Supplement: Additional file 1 — PKAR1/Alexa594-cAMP colocalization in the normal brain. FIGURE S1. A section from normal brain (case 4) is shown, 100× objective, bar = 10 μm. A. immunohistochemistry for PKA R1, revealed with Alexa594 (red)-conjugated secondary antibody. B. the same section was then incubated with Alexa488 (green)-tagged cAMP. The arrows indicate two autofluorescent cells. C. Merge of A and B: the specific green signal overlaps with red signal. [file 1471-2407-10-141-S1.TIFF]
